# Pituitary apoplexy presenting as isolated third cranial nerve palsy: case series

**DOI:** 10.1093/jscr/rjac386

**Published:** 2022-08-23

**Authors:** Ramesh Shrestha, Suresh Bishokarma, Sushil Rayamajhi, Sunita Shrestha, Saurav Lamichhane, Pratyush Shrestha, Suraj Thulung

**Affiliations:** Department of Neurosurgery, Upendra Devkota Memorial National Institute of Neurological and Allied Sciences, Kathmandu 44600, Nepal; Department of Neurosurgery, Upendra Devkota Memorial National Institute of Neurological and Allied Sciences, Kathmandu 44600, Nepal; Department of Neurosurgery, Upendra Devkota Memorial National Institute of Neurological and Allied Sciences, Kathmandu 44600, Nepal; Department of Neurosurgery, Upendra Devkota Memorial National Institute of Neurological and Allied Sciences, Kathmandu 44600, Nepal; Department of Neurosurgery, Upendra Devkota Memorial National Institute of Neurological and Allied Sciences, Kathmandu 44600, Nepal; Department of Neurosurgery, Upendra Devkota Memorial National Institute of Neurological and Allied Sciences, Kathmandu 44600, Nepal; Department of Neurosurgery, Upendra Devkota Memorial National Institute of Neurological and Allied Sciences, Kathmandu 44600, Nepal

## Abstract

Pituitary apoplexy (PA) is caused by a sudden increase in pressure in the pituitary region due to acute hemorrhage, infarction or necrosis. PA can also be caused by restricting blood supply to the nerve due to compression of the internal carotid artery. Acute third cranial nerve palsy (third CN) secondary to PA is a rare medical emergency caused by bleeding within a growing mass within the sella turcica. We presented two cases of PA with isolated third CN palsy treated with transsphenoidal pituitary decompression. PA is therefore an important differential diagnosis to consider in patients with isolated third nerve palsy. The prognosis for isolated third nerve palsy in PA appeared successful, with variable recovery from medical and surgical intervention.

## INTRODUCTION

Pituitary apoplexy (PA) is a sudden neurological deficit caused by an expanding mass within the sella turcica, which may develop from pituitary hemorrhage, infarction or both, or from a pre-existing pituitary tumor such as an adenoma [1]. As a result, it is associated with a clinical constellation of symptoms such as sudden onset headache, visual disturbances and ophthalmoplegia due to third, fourth or sixth cranial nerve (CN) involvement [2]. PA has a prevalence of ~6.2 cases per 100 000 population and an annual incidence of 0.17 cases per 100 000 population [3, 4]. Isolated third CN palsy is a rare symptom of PA [5]. We presented the case reports of two patients who had acute isolated third CN palsy secondary to pathologically proven PA with variable healing processes.

## CASE REPORT

### Case 1

A 56-year-old hypertensive man presented to our emergency department with a chronic frontotemporal headache that had been worsening for 2 days and was associated with acute left ptosis without blurred vision or diplopia. There had been no loss of consciousness or seizures. On examination, his Glasgow coma scale (GCS) was 15/15. The right pupil was 3 mm light reactive, whereas the left was 4 mm non-responsive. Visual acuity and visual field circumference were normal in both eyes.

**Figure 1:**
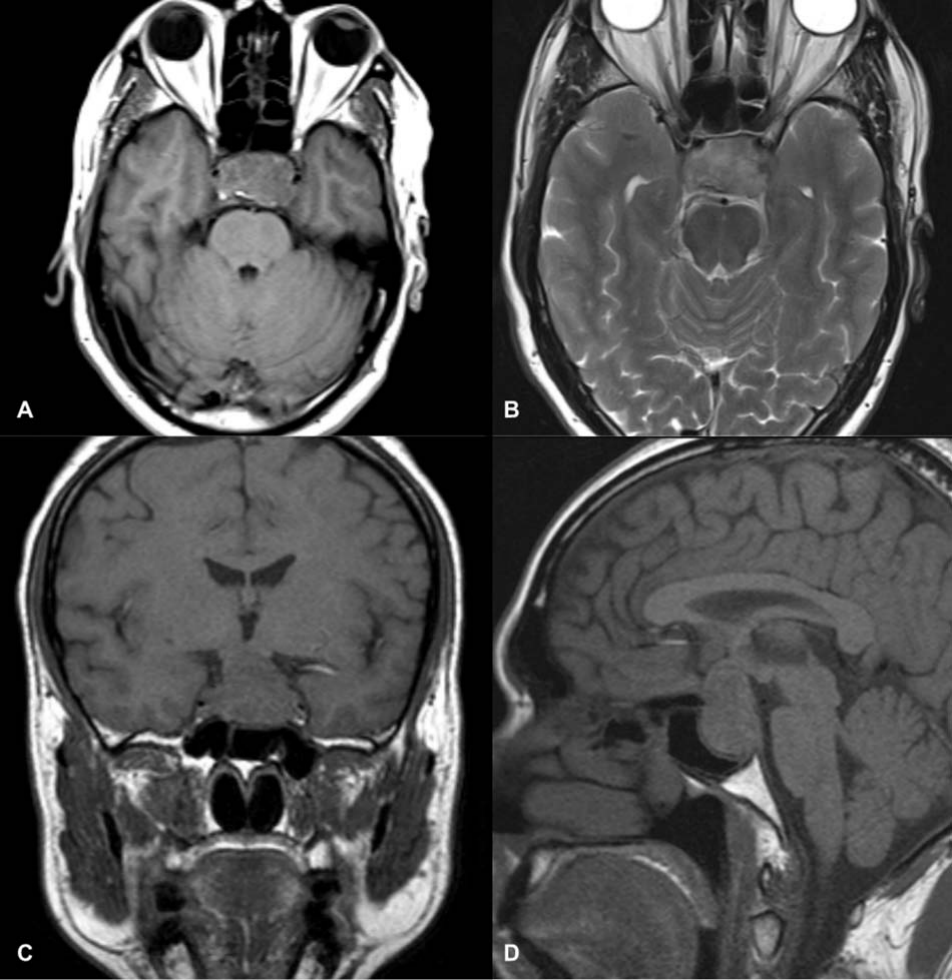
MRI images of axial T1 (**A**), axial T2 (**B**), coronal T1 (**C**), and sagittal T1-weighted images (**D**) showing a 3 cm × 2.9 cm × 1.8 cm sized suprasellar lesion displacing optic chiasma superiorly and laterally causing a mass effect on bilateral cavernous sinuses (Left>Right) with patchy areas of high signal on T1-weighted images and signal void on T2-weighted images suggestive of hemorrhage.

Magnetic resonance imaging (MRI) (axial T1, axial T2, coronal T1 and sagittal T1-weighted images) revealed a sellar and suprasellar lesion measuring 3 × 2.9 × 1.8 cm with patchy areas of high signal on T1-weighted images and signal void on T2-weighted images suggestive of hemorrhage. The suprasellar component of the lesion was displacing optic chiasma superiorly and the sellar component was causing sellar expansion with mass effect on bilateral cavernous sinuses (left>right); right internal carotid artery (ICA) had a contact angle of 90°, whereas left ICA had >90° (Fig. 1).

Preoperative hormone analysis showed normal levels of thyroid-stimulating hormone (TSH), growth hormone (GH), insulin-like growth factor (IGF), follicle-stimulating hormone (FSH), prolactin, free thyroxine, cortisol and luteinizing hormone (LH) levels.

**Figure 2:**
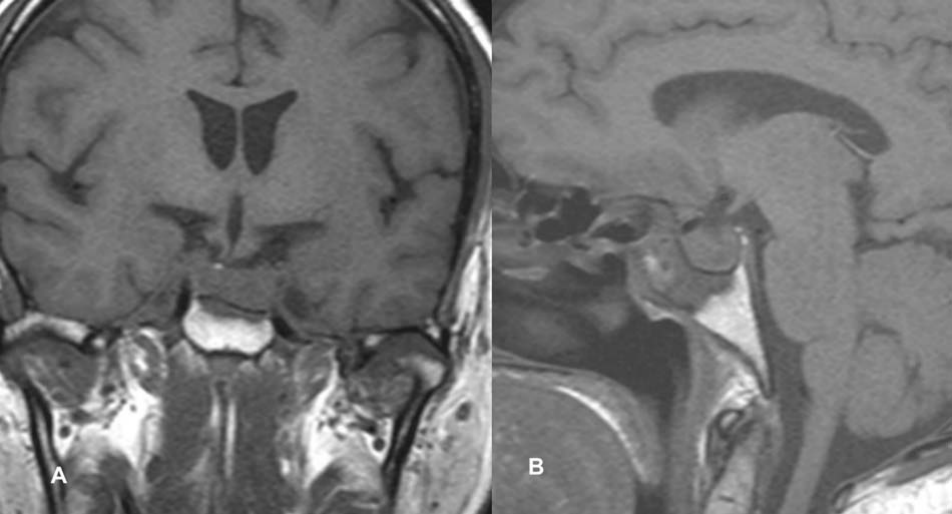
Follow-up MRI imaging of case 1 showed the sellar, suprasellar residual lesion measuring 1.9 × 1.5 × 1.1 cm without a significant mass effect on cavernous sinus.

PA was diagnosed when radiographic findings showed acute pituitary hemorrhage and subsequent pituitary adenectomy was planned, but surgery was delayed up to 13 days because of non-critical coronary artery disease detected on coronary angiography. When his condition stabilized, he underwent surgery. The tumor was soft to firm and a safe maximal resection was performed. Hemostasis was achieved and sella packed with fat harvested from the subcutaneous fat of the abdomen. Postoperatively, he suffered transient diabetes insipidus, which was treated with desmopressin injections and resolved by day 5 postoperatively. He was then discharged on the 8th postoperative day. At the time of discharge, he had residual ptosis, which gradually improved over 2 weeks based on clinical assessment at the outpatient visit. He fully recovered from ptosis over a month. Postoperative imaging performed at 6 weeks showed a residual tumor measuring 1.9 × 1.5 × 1.1 cm with no significant mass effect on the cavernous sinus; therefore, he was referred to an oncologist for postoperative radiotherapy (Fig. 2).

### Case 2

A 50-year-old gentleman presented with painful ptosis of the left eye for 4 days with no signs of raised intracranial pressure, seizure or loss of consciousness. Clinically, GCS was 15/15. The right pupil was 2 mm reactive to light, whereas the left pupil was 4 mm sluggishly reactive, with no signs of meningism.

**Figure 3:**
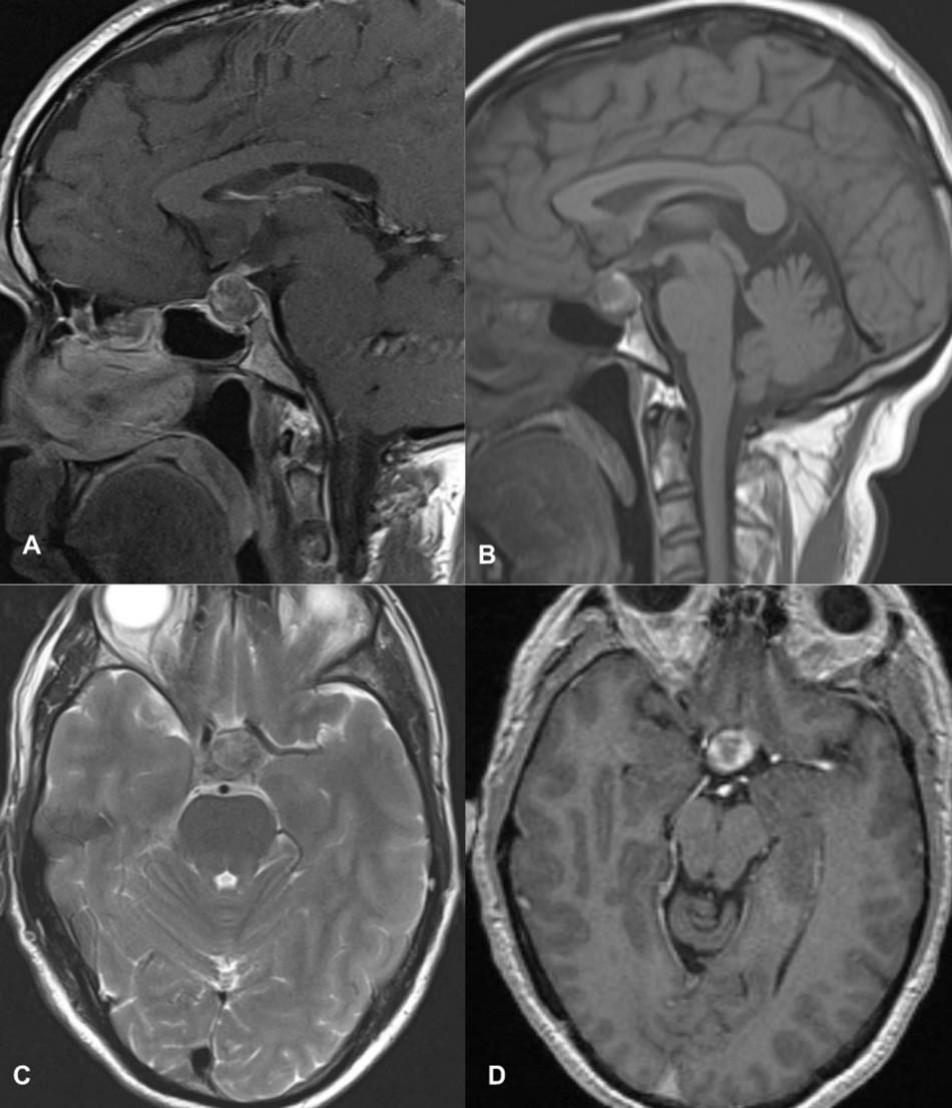
MRI images of post-contrast sagittal T1 (**A**), sagittal T1 (**B**), axial T2 (**C**), and post-contrast axial T1-weighted images (**D**) showing a 2 cm × 1.6 cm × 1.6 cm sized sellar lesion arising from the pituitary fossa extending superiorly in the suprasellar region and laterally causing a mass effect on the left cavernous sinus with intrinsic high T1 signal in the periphery and signal void on T2-weighted images suggestive of hemorrhage.

MRI imaging showed a 2 × 1.6 × 1.6 cm sized sellar lesion arising from the pituitary fossa with an intrinsic high T1 signal in the periphery, extending superiorly in the suprasellar region and laterally, causing a mass effect on the left cavernous sinus. A signal void was seen in T2-weighted images suggestive of hemorrhage (Fig. 3).

A preoperative hormonal evaluation was performed, which showed normal levels of TSH, GH, IGF, FSH, prolactin, free thyroxine, cortisol and LH.

**Figure 4:**
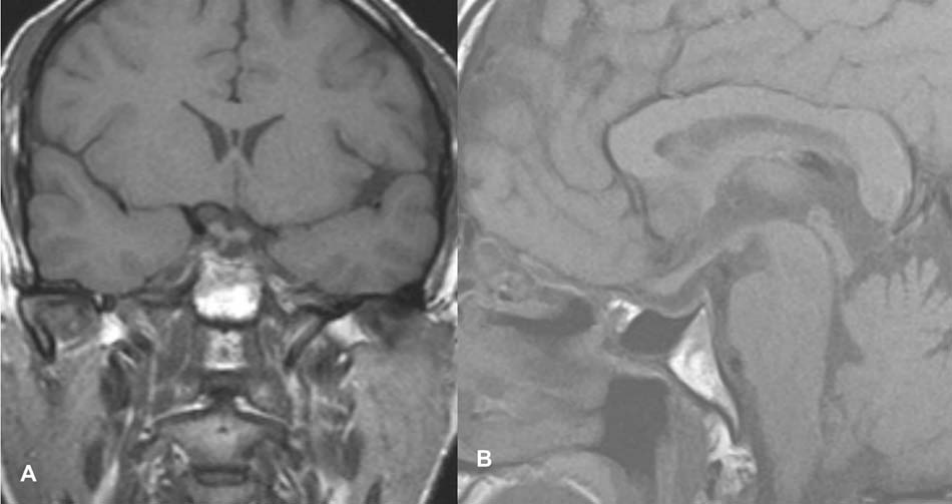
Follow-up imaging of case 2 showed no evidence of a residual lesion in sella turcica.

The patient underwent transsphenoidal pituitary adenectomy 3 days after admission. Intraoperatively, soft tumors with hemorrhagic components were seen. Hemostasis was achieved and the sella was packed with fat. The patient was managed in the ICU for 2 days and discharged on the 8th postoperative day. The ptosis gradually improved within 2 days of surgery, and during the 2-week follow-up in the outpatient clinic, the ptosis recovered completely. Follow-up imaging showed no evidence of a residual lesion in sella turcica (Fig. 4).

## DISCUSSION

PA is a rare medical emergency caused by a growing mass within the sella turcica, secondary to pituitary hemorrhage or infarction, or within a pre-existing pituitary adenoma [1]. We reported on two patients who presented with acute third CN palsy and who were later diagnosed with PA based on histopathological findings.

According to recent epidemiological studies, it has an annual incidence of 0.17 per 100 000 people and a prevalence of ~6.2 cases per 100 000 people [3, 4, 6]. In >80% of cases, PA is the first presentation of an underlying pituitary tumor and is characterized by rapid onset headache, which is usually the most common initial symptom, along with visual disturbances, ocular paralysis and acute onset hypopituitarism [2]. More than half of PA patients experience vision problems caused by a sudden increase in tumor mass, resulting in compression of the optic sacrum or optic nerves from an upward tumor extension [7]. The age- and sex-adjusted annual incidence of acquired third nerve palsy was 4.0 per 100 000 people [6].

A third CN palsy as a presentation of PA is rarely present [5]. It is characterized by a combination of ptosis, impaired pupillary constriction and ophthalmoparesis, depending on where the lesion is between the midbrain and orbit. Microvascular ischemia caused by diabetes or systemic hypertension, inflammatory processes such as giant cell arteritis, trauma, compressive mass lesions, i.e. intracranial tumors, or aneurysms are all major causes of third CN palsy [8].

The differential diagnosis of third CN palsy can be narrowed down by the presence or absence of accompanying pain and pupillomotor impairment. Compression lesions, particularly aneurysms, result in pupillary involvement, and isolated complete third nerve palsy with normal pupillary function characterizes ischemic lesions [8]. Compression lesions, such as posterior communicating artery aneurysms or tumors, usually involve both the superficial and deep fibers, resulting in oculomotor deficits and pupillary dysfunction. However, while ischemic lesions usually affect the central core of the nerve that controls oculomotor function, the superficial parasympathetic fibers are usually unaffected (pupil spares third nerve palsy) [9].

Several explanations have been proposed to explain why PA causes third nerve palsy. Because the oculomotor nerve lies horizontally in the same plane as the pituitary gland in the lateral wall of the cavernous sinus, it is often the first CN involved in the lateral spread of a pituitary tumor or hematoma [10]. Because CNs are sensitive to pressure, a sudden increase in pressure in the pituitary region due to acute hemorrhage, infarction or pituitary necrosis can be transmitted to the third CN with relative ease [11]. Acute third nerve palsy can also be caused by a reduction in the blood supply to the nerve due to compression of the vasa nervorum emanating from the ICA [12].

Imaging of our patient showed a sellar and suprasellar mass with an acute hemorrhagic component and a mild mass effect on the left cavernous sinus. As a result of this, in our patient, there is a high probability of pressure on the third CN or impairment of the vascular supply of the nerve due to compression of the vasa nervorum from the ICA as the cause of the third CN palsy. To prevent acute adrenal insufficiency, patients with PA require intravenous steroids until surgery. The definitive treatment for PA is neurosurgical decompression via a transsphenoidal approach. Early decompression can partially or fully restore pituitary and third CN function [13].

Patients who underwent surgery had better clinical improvement and endocrinological results since a viable tumor component could be removed from constrained areas such as the cavernous sinus [14]. However, in the recent study by Sipos *et al*. [15], conservative care is preferable to immediate neurosurgical intervention if the ophthalmoplegia is improving or stable. According to the literature, when visual acuity defects do not improve, surgery is performed in <7 days. Furthermore, this reduces the need for ongoing hormonal replacement [16].

Tumor size regression is directly related to better ophthalmologic and endocrinologic outcomes [17, 18]. Early intervention may be able to reverse or partially reverse pituitary function while preserving the third CN function [13]. The poor surgical outcomes could result from preoperative endocrine abnormalities [19]. A multidisciplinary approach, outcome reporting and institutional experience all have an impact on the outcome. Hence, before recommending evidence-based guidelines, a prospective study is required [20]. In order to determine the best immediate management with neurosurgical decompression via a transsphenoidal approach for PA, authors have suggested that patients with large tumors (>11.67 mm) or higher Knosp should be evaluated on a case-by-case basis [21].

Our patients underwent transsphenoidal neurosurgical decompression and pathological examination, confirming the diagnosis of PA. After the operation, the third CN palsy was completely resolved. The first patient underwent delayed decompression due to cardiac problems where recovery of third nerve function was delayed compared with the early decompression in the second patient. This finding may favor early decompression to restore the function of the third nerve palsy.

## CONCLUSION

PA is an essential differential diagnosis in patients with isolated third nerve palsy. The prognosis for isolated third nerve palsy in PA appeared successful with variable recovery with medical and surgical interventions.
